# Comparison of Acquired Activated Protein C Resistance, Using the CAT and ST-Genesia^®^ Analysers and Three Thrombin Generation Methods, in APS and SLE Patients

**DOI:** 10.3390/jcm11010069

**Published:** 2021-12-23

**Authors:** Maria Efthymiou, Philip J. Lane, David Isenberg, Hannah Cohen, Ian J. Mackie

**Affiliations:** 1 Haemostasis Research Unit, Department of Haematology, University College London, London WC1E 6HX, UK; p.lane@nhs.net (P.J.L.); hannah.cohen@ucl.ac.uk (H.C.); i.mackie@ucl.ac.uk (I.J.M.); 2 Department of Rheumatology, University College London Hospitals NHS Foundation Trust, London NW1 2PG, UK; d.isenberg@ucl.ac.uk; 3 Centre for Rheumatology, Division of Medicine, University College London, London WC1E 6JF, UK; 4 Department of Haematology, University College London Hospitals NHS Foundation Trust, London NW1 2PG, UK

**Keywords:** antiphospholipid syndrome, systemic lupus erythematosus, blood coagulation test, activated protein C resistance, thrombomodulin, activated protein C

## Abstract

Background: Acquired activated protein C resistance (APCr) has been identified in antiphospholipid syndrome (APS) and systemic lupus erythematosus (SLE). Objective: To assess agreement between the ST-Genesia^®^ and CAT analysers in identifying APCr prevalence in APS/SLE patients, using three thrombin generation (TG) methods. Methods: APCr was assessed with the ST-Genesia using STG-ThromboScreen and with the CAT using recombinant human activated protein C and Protac^®^ in 105 APS, 53 SLE patients and 36 thrombotic controls. Agreement was expressed in % and by Cohen’s kappa coefficient. Results: APCr values were consistently lower with the ST-Genesia^®^ compared to the CAT, using either method, in both APS and SLE patients. Agreement between the two analysers in identifying APS and SLE patients with APCr was poor (≤65.9%, ≤0.20) or fair (≤68.5%, ≥0.29), regardless of TG method, respectively; no agreement was observed in thrombotic controls. APCr with both the ST Genesia and the CAT using Protac^®^, but not the CAT using rhAPC, was significantly greater in triple antiphospholipid antibody (aPL) APS patients compared to double/single aPL patients (*p* < 0.04) and in thrombotic SLE patients compared to non-thrombotic SLE patients (*p* < 0.05). Notably, the ST-Genesia^®^, unlike the CAT, with either method, identified significantly greater APCr in pregnancy morbidity (median, confidence intervals; 36.9%, 21.9–49.0%) compared to thrombotic (45.7%, 39.6–55.5%) APS patients (*p* = 0.03). Conclusion: Despite the broadly similar methodology used by CAT and ST-Genesia^®^, agreement in APCr was poor/fair, with results not being interchangeable. This may reflect differences in the TG method, use of different reagents, and analyser data handling.

## 1. Introduction

The anticoagulant protein C pathway plays a central role in the regulation of coagulation and in the active cross-talk between the inflammation and coagulation systems. The physiological proteolytic activation of protein C by thrombin occurs on the endothelial surface and involves two membrane receptors, thrombomodulin (TM) and endothelial protein C receptor. The binding of thrombin to TM shields the procoagulant exosite I of thrombin and facilitates protein C activation [1]. Activated protein C (APC) exerts its anticoagulant effects by proteolytic inactivation of factor Va and factor VIIIa, with protein S acting as a cofactor in these reactions^1^. In addition to its anticoagulant properties, activated protein C also exerts cytoprotective and anti-inflammatory effects, including inhibition of leukocyte chemotaxis, reduction in expression of pro-inflammatory cytokines, and expression of adhesion molecules [2]. Resistance to the anticoagulant actions of activated protein C (referred to as activated protein C resistance, APCr) either heritable (i.e., caused by factor V Leiden) [3,4] or acquired, has been shown to be associated with hypercoagulability and an increased risk of thromboembolic events [5,6,7].

Acquired APCr, assessed using the thrombin generation (TG) system, which provides a global assessment of coagulation function, in the presence and absence of APC that enables assessment of the function of the protein C system, was shown to be associated with thrombosis in antiphospholipid antibody (aPL) positive patients [8]. Using the calibrated automated thrombogram (CAT), APCr to both exogenous APC and to activation of endogenous plasma protein C using Protac^®^ was shown to be greater and associated with a more severe thrombotic phenotype in antiphospholipid syndrome (APS) patients with previous venous thromboembolism [9]. Similarly, increased APCr, using the same system, and independently of criteria aPL was also demonstrated in systemic lupus erythematosus (SLE) patients [10]. In spite of the numerous attempts to standardise tests with the CAT system, the methodology is characterised by high inter- and often intra-laboratory variation, lack of standardisation, clinical validation and absence of quality controls [11,12,13,14,15,16]. More recently, it was demonstrated that with the use of standardised methodology, use of commercial reference plasma and quality controls for validation of each run of measurement, the ETP based APCr assay can be reproducible, sensitive and validated with excellent inter-experiment precision [17].

The ST-Genesia^®^ (Stago, France) is a new, fully automated TG analyser with customised reagents sensitive to procoagulant and anticoagulant protein deficiencies. In comparison to the CAT system, it offers normalisation of each TG parameter to a reference plasma for each test performed and has been designed to offer enhanced reproducibility and standardisation with the use of dedicated calibrators and controls, with the aim of reducing inter-laboratory and inter-assay variability [18,19,20,21]. However, the calibration used to obtain thrombin concentration from the fluorescent signal differs between the two TG systems.

The aims of this prospective cross-sectional study were to a) evaluate the prevalence of APCr in APS patients and SLE patients compared to non-APS/SLE thrombotic controls using the ST-Genesia^®^ system in the presence/absence of TM and b) to compare APCr with the CAT system (using recombinant human APC (rh-APC) and Protac^®^) to establish if both systems can detect APCr observed in APS and SLE patients.

## 2. Methods

### 2.1. Patients and Samples

All patients recruited in this study fulfilled the relevant international disease classification criteria for either APS [22] or SLE [23]. Disease activity using the British Isles Lupus Assessment Group (BILAG)-2004 index [24] and the SLE disease activity index-2000 (SLEDAI-2K) [25] were recorded for all patients with SLE. BILAG categories were converted into numbers according to the 2010 coding scheme [26]. Patients with APS and thrombotic controls were receiving warfarin anticoagulation for at least three months since the thromboembolic event prior to being recruited. In this cross-sectional study, we tested for APCr the following patients: 105 APS patients with no other autoimmune diseases (83 thrombotic, venous and/or arterial thrombosis, and 23 with only pregnancy morbidity; PM), 53 SLE patients (16 with APS and 37 with no thrombosis), and 36 non-APS thrombotic controls. Seventy-five healthy normal controls were also recruited and used to establish cut off values for the assays. APCr with the CAT system was previously reported for 30 thrombotic APS, 20 non-thrombotic controls [9] and for 53 SLE patients [10].

Written informed consent was obtained from all subjects in accordance with the Declaration of Helsinki. Ethical approval was granted by the Research Ethics Committee NREC (reference: 13/EM/0150) and from the Research and Development office at UCLH (reference: 13/0030). Patients (APS, SLE and thrombotic controls) were excluded if they had heritable thrombophilia (factor V Leiden or the G20210A prothrombin gene mutation, antithrombin, protein S or protein C deficiency), a history of malignancy or myeloproliferative neoplasms. Patients and NC were also excluded if they were receiving estrogen preparations (combined oral contraceptives or hormone replacement therapy) or were pregnant. All samples were collected between 2017 to 2020 and were stored for a maximum of three years prior to their use.

Clinical data were collected from medical records and included demographics, general disease characteristics over time, history of thrombotic events and medication. Antiphospholipid antibodies had been routinely assessed in the hospital laboratory with diagnostic procedures and assessment of aPL profile and status at the time of sampling performed in accordance with international consensus criteria and national guidelines [22,27,28]. A positive aPL profile was defined as the presence of at least one aPL type, confirmed by repeat assessment at least 12 weeks apart with antibody levels (β2 Glycoprotein I antibodies and cardiolipin antibodies) exceeding the 99th percentile of the laboratory reference range and lupus anticoagulant was positive/negative [22].

Venous blood was collected using a 21-gauge butterfly needle, with minimal venous stasis, into 5 mL Vacutainer^®^ tubes (Becton Dickinson, Plymouth, UK) containing 0.105 M citrate. Platelet poor plasma was prepared within two hours of collection by double centrifugation at ambient temperature (2000× *g* for 15 min) and stored in aliquots at −80 °C. Immediately prior to analysis, the samples were thawed in a water bath at 37 °C. 

APCr using the ST-Genesia^®^ analyser: TG was investigated according to the manufacturer’s recommendations using the STG-ThromboScreen reagent, in the absence (−) and presence (+) of TM (Stago, Asnières sur Seine, France). The reagent contains a mixture of phospholipids and human TF at a medium picomolar concentration, referred to infra as “intermediate picomolar TF concentration (concentration not disclosed by manufacturer). Each batch of both reagents is adjusted by the manufacturer to obtain the desired TG profile (“reagents manufacturer undisclosed data”). The reagent TM concentration in the reagent is sufficient to inhibit 50% of the ETP obtained in normal pooled plasma in the absence of TM (final TM concentration is not disclosed by Stago). The assay contained three levels of quality control, low, normal, and high TM resistance and a reference plasma for parameter normalisation. In the presence of both reagents, TG was triggered by the CaCl_2_ contained in a combined reagent with the fluorogenic substrate. The intra- and inter-assay coefficient of variation using pooled normal plasma for the ST-Genesia^®^ system in the presence of TM was: 1.1%, 0.9% and 0.9%; and 2.1%, 3.0%, and 4.9% for lag time, ETP, and peak thrombin respectively.

APCr using the CAT analyser: Resistance to exogenous APC was determined using 5 nM recombinant (rh) APC, and to activation of endogenous protein C using 0.1 units/mL Protac^®^, an enzyme that converts protein C into APC (Pentapharm AG, Basle, Switzerland) using the CAT machine with the PPP reagent (5 pM tissue factor) as previously described [9,10].

APCr assays using the CAT and the ST-Genesia^®^ systems in samples from patients on warfarin anticoagulation were performed by mixing patient plasma 50:50 with pooled normal plasma to correct any factor deficiencies induced by anticoagulation, as described by us and others [9,29,30].

APCr was expressed as % inhibition of endogenous thrombin potential (ETP), ETP (in nmol/L·min: area under the thrombin time concentration curve) where ETP is the amount of thrombin formed in vitro in a clotting reaction and reflects the in vivo capacity of an individual to generate thrombin.

The % inhibition was calculated by (the result of TG parameter (ETP) in the absence of TM minus ETP in the presence of TM)/(ETP absence of TM) × 100 and for the CAT assays for rhAPC and Protac^®^ as previously described [9,30]. Greater APCr is defined as % inhibition of ETP below the ninety-ninth centile of 75 NC; rhAPC (56%); Protac^®^ (63%), −/+TM (49%). APCr is increased as the %inhibition of ETP decreases.

### 2.2. Statistical Analysis

Results are expressed as median with 95% confidence intervals (CI). Comparisons were made using the Mann–Whitney test or the Wilcoxon signed-rank test when appropriate. Results are reported as median and 95% confident intervals. Statistical comparisons of the results obtained with the different experimental conditions within and between different patient groups were performed using paired *t*-tests. The degree of agreement between methods was assessed categorically according to the presence or absence of APCr below the ninety-ninth centile in NC, using the kappa (κ) coefficient, where κ < 0 shows no; <0.20 poor; 0.2–0.40 fair; 0.41–0.60 moderate; 0.61–0.80 good; and 0.81–1.00 very good agreement [31]. The Bland-Altman method was used to evaluate the agreement between methods by constructing 95% limits of agreement. Fisher’s exact test was used to study associations. A *p*-value of <0.05 was considered to be significant. Statistical analysis was performed using Graph Pad 8.0.

## 3. Results

### 3.1. Patients

Characteristics, clinical features and medication for patients with APS and thrombotic controls are presented in Table S1 and for patients with SLE in Table S2. There were no major differences between the patient groups in terms of demographics, SLE clinical features and disease activity at the time of sample collection. According to the APS classification (categories: I, IIa, IIb, and Iic, based on Miyakis et al., 2006) [22], 64/105 APS patients were category I (more than one laboratory criteria present; 45 of whom were double and 19 triple aPL positive); 16 were category IIa (LA alone), 11 were category IIb (presence of aCL alone); 14 patients were category IIc (presence aβ_2_GPI alone). Out of the 36 SLE patients with aPL, 29 were category I (16 double and 13 triple aPL positive), four category IIa, two category Iib, and one category IIc (Table S2).

**Figure 1:**
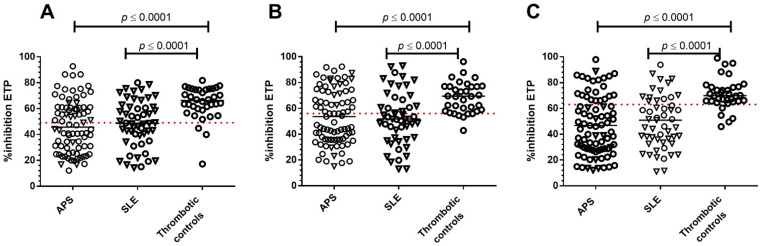
Scatter plot graph of activated protein C resistance (APCr) in APS, SLE patients and thrombotic controls: (**A**) ST-Genesia^®^ in the presence of thrombomodulin (TM); (**B**). CAT analyser in the presence of recombinant activated protein C (rhAPC) and (**C**). Protac^®^. The horizontal broken line indicates the ninety-ninth centile of NC for each method. Median values in each patient group are indicated. APS patients include thrombotic and pregnancy morbidity (PM), and SLE patients include both thrombotic and non-thrombotic. Patients on warfarin anticoagulation are represented with an open circle symbol (○), and patients on no anticoagulation treatment with an inverted triangle (▽).

APCr with ST-Genesia^®^: Percent (%) inhibition of ETP with the ST-Genesia^®^ in the presence of TM was significantly lower in APS and SLE patients compared to thrombotic controls (*p* < 0.0001 for both). No differences were observed between APS and SLE patients (Figure 1), with no significant differences in % inhibition of ETP between methods and reagents in any of the patient groups. Using the ST-Genesia^®^, APCr values (below the established normal cut off) were identified in 53.8% APS, 50% SLE patients and 8.3% thrombotic controls. Using the CAT analyser, APCr with rhAPC was identified in 57.5% APS, 59.3% SLE patients, and 16.7% thrombotic controls; and APCr with Protac^®^ in 63.2% APS, 70.4% SLE, and 13.9% thrombotic controls (Table 1). Subgroup-analysis of the patients that were receiving warfarin anticoagulation revealed that APCr was significantly different between thrombotic controls and thrombotic APS patients (*p* < 0.0001 for all three methods) as was also between thrombotic controls and thrombotic SLE patients (ST-Genesia^®^; *p* = 0.03, CAT rhAPC; *p* = 0.003, Protac^®^; *p* < 0.0001).

**Figure 2:**
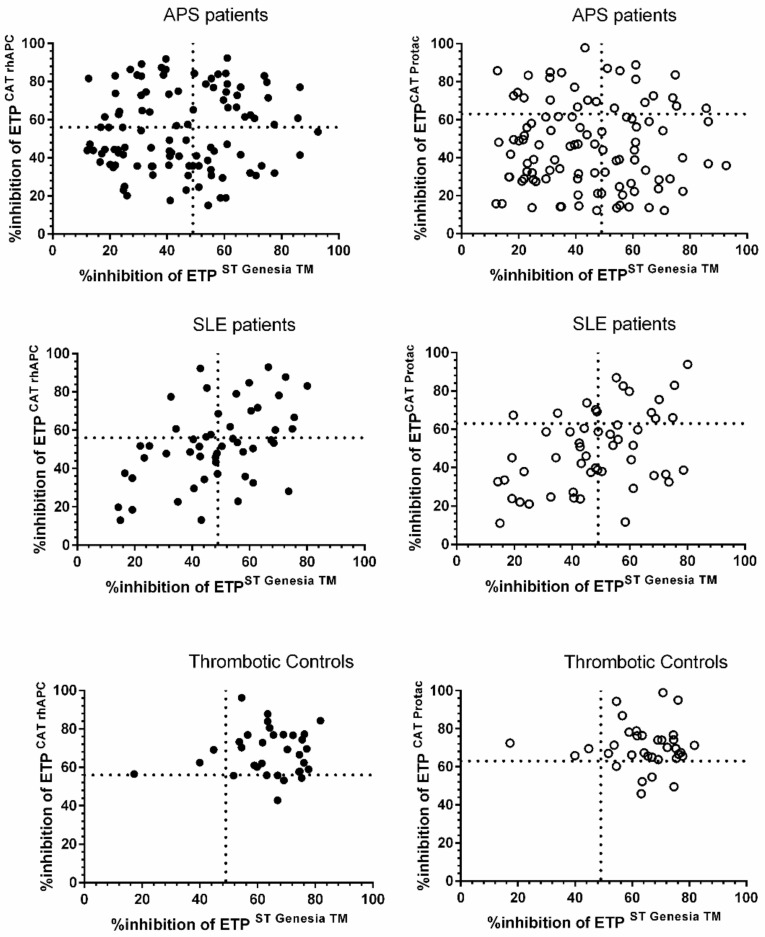
Agreement in APCr between the ST-Genesia^®^ in the presence of TM and the CAT analyser with rhAPC (**left** panel) and Protac^®^ (**right** panel) in APS, SLE patients and thrombotic controls. Vertical broken line represents the cut off value for APCr with the ST-Genesia^®^ (49%), and the horizontal dotted line in the left panel represents the APCr cut off value of 56% for the CAT with rhAPC, and in the right panel, the APCR cut off value of 63% for the CAT with Protac^®^. All cut-offs were defined as values below the nintey-ninth centile of NC.

However, agreement between the ST-Genesia^®^ and the CAT analyser (using either rhAPC or Protac^®^) in identifying patients with APCr was only poor to fair for APS and SLE patients, respectively (Figure 2, Table 1). In thrombotic controls, there was no agreement between the two analysers (Figure 2, Table 1), but the number of patients with APCr below the established cut off was small, and most of these had borderline results.

Bland-Altman analysis showed consistently lower APCr values with the ST-Genesia^®^ compared to the CAT analyser (for both rhAPC and Protac^®^) with a small degree of bias, but no particular trends and with varying APCr levels for all patient groups (Figure S1).

### 3.2. Patients with APS

Patients with APS were further stratified (a) according to clinical phenotype: into thrombotic (venous and/or arterial) and PM patients, and (b) according to aPL status (single, double triple aPL positive).

**Figure 3:**
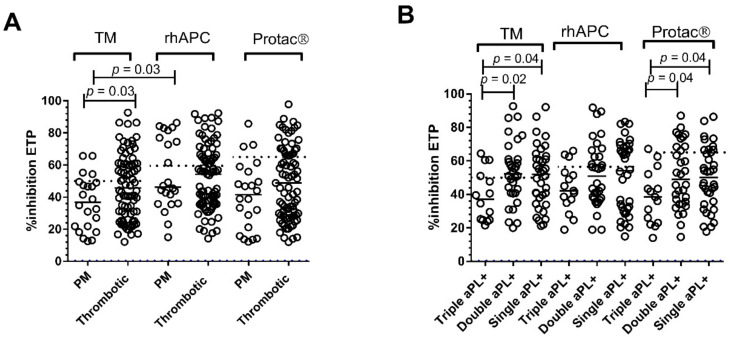
APCr with TM, rhAPC, and Protac^®^ in APS patients stratified according to (**A**). clinical phenotype: thrombotic and pregnancy complications (PM) and (**B**). antiphospholipid antibody status. Dotted lines indicate the cut off for each method used.

Percent inhibition of ETP with the ST-Genesia^®^ was significantly lower in PM compared to thrombotic APS patients (*p* = 0.03), but it failed to reach significance with the CAT analyser with either rhAPC or Protac^®^ (Figure 3).; agreement between the three methods was poor to fair. In APS patients with PM, % inhibition of ETP with the ST-Genesia^®^ was significantly lower compared to the CAT analyser with rhAPC, (*p* = 0.03) but not with Protac^®^ (Figure 3A). No differences were observed in median APCr between the three methods in thrombotic APS patients (Figure 3A, Table 2) or between venous and arterial APS patients (data not shown).

Agreement in APCr between the ST-Genesia^®^ and the CAT with rhAPC was fair in thrombotic and poor in PM APS patients while with Protac^®^ was poor for both clinical subgroups (Figure S2, Table 2).

APCr was identified in 66.6% of triple aPL + APS patients with the ST-Genesia^®^ compared to 80% with the CAT analyser with rhAPC and 93.3% with Protac^®^. Comparable prevalence of APCr for double and single aPL + APS patients with the two analysers was observed (Table 2).

In triple aPL positive APS patients APCr values with the ST-Genesia^®^ and the CAT analyser with Protac^®^ were significantly greater compared to double and single aPL positive APS patients. (Figure 3B, Table 2).

Moderate agreement was observed in the triple aPL+ APS patients between the Genesia^®^ using TM and the CAT using rhAPC or Protac^®^ but not in double or single aPL positive APS patients, which showed fair and poor agreement between either method (Figure S2, Table 2).

Bland-Altman analysis showed a trend towards lower APCr values with the ST-Genesia^®^ when compared to the CAT with rhAPC in both thrombotic and PM APS (Figure S3A,B) and in all aPL positive APS groups (Figure S3).

### 3.3. Patients with SLE

**Figure 4:**
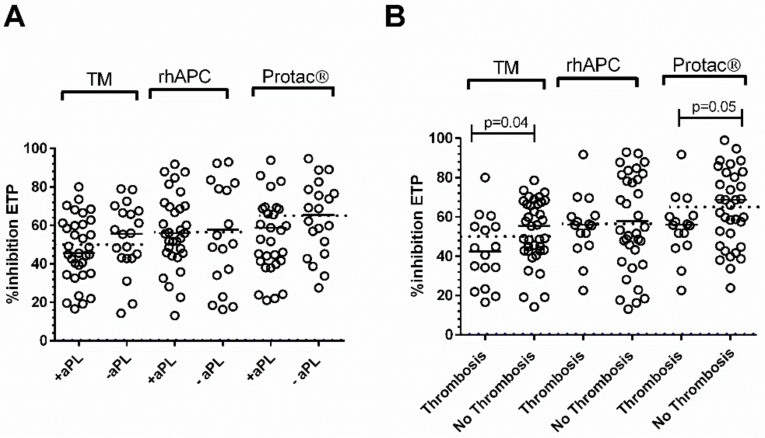
APCr with TM, rhAPC, and Protac^®^ in patients with SLE stratified according to (**A**) aPL status and (**B**) thrombotic history. Dotted lines indicate the cut off for each method used.

Patients with SLE were further stratified according to aPL status (positive and negative) and thrombotic history (with and without thrombosis). There were no differences in APCr values with either of the analysers between aPL positive and negative SLE patients (Figure 4A).

However, % inhibition of ETP with the ST-Genesia^®^ and the CAT analyser with Protac^®^ was significantly lower in those with thrombosis compared to those without (TM; *p* = 0.04, Protac^®^; *p* = 0.05). There were no differences in APCr values assessed with any of the three methods between thrombotic APS patients and SLE patients with APS. 

Lower % inhibition of ETP with the ST-Genesia^®^ and the CAT analyser with either rhAPC or Protac^®^ was identified in between 45–53% of aPL positive and negative patients.

In SLE patients, only poor to fair agreement in APCr was seen between the two analysers and between the three methods (Figure S4, Table 2).

Bland–Altman analysis showed a trend towards higher APCr values with the CAT analyser (rhAPC or Protac^®^) compared to values obtained with the ST-Genesia^®^ for all the different groups of SLE patients tested (Figure S5).

## 4. Discussion

This study reports on the novel comparison of two TG analysers, the ST-Genesia^®^ and CAT, using three different TG methods in assessing APCr in patients with APS, SLE, and in thrombotic controls.

Percent inhibition of ETP with the ST-Genesia^®^ in the presence of TM was significantly lower in overall APS and SLE patients compared to thrombotic controls. Sub group-analysis of anticoagulated patients also revealed that APCr with all three methods was significantly greater in thrombotic APS and thrombotic SLE patients when compared to thrombotic controls. We demonstrated that regardless of the TG method used, agreement in identifying APCr between the two analysers was poor in APS patients (≤65.9%, k coefficient: ≤0.20) and fair in SLE patients (≤68.5%, k coefficient: ≥0.29). No agreement was observed in thrombotic control patients, probably due to the small number of patients with APCr with only borderline abnormality.

When APS and SLE patients were further stratified according to clinical phenotype and aPL status, we observed that APCr with both the ST Genesia and the CAT using Protac^®^, but not with the CAT using rhAPC, was significantly greater in triple aPL APS patients compared to double/single aPL patients and in thrombotic SLE patients compared to non-thrombotic SLE patients.

A novel observation of our study was that the ST-Genesia^®^ identified significantly less % inhibition of ETP in PM compared to thrombotic APS patients, which was not identified using the CAT analyser with either of the two methods used. APCr values were consistently higher with the CAT analyser compared to ST-Genesia^®^ in all patient groups, and results were not interchangeable.

APCr using the TG can be assessed by either investigating the downstream effects of APC, using exogenous APC or by assessing the integrity of the mechanism of endogenous protein C activation, using either TM or Protac^®^, which can highlight differences in the development of APCr [32]. Previous APCr studies in APS patients mainly employed exogenous APC and the CAT analyser and demonstrated a clear association between increased APCr and thrombotic events [30,33]. Our group extended the APCr studies in APS patients with the CAT analyser by using Protac^®^ to assess activation of endogenous protein C, showing that APCr with Protac^®^ compared to rhAPC was associated with a severe thrombotic phenotype in venous thrombosis APS patients^9^. Previous studies have also confirmed that APCr is frequently present in SLE using the CAT analyser and exogenous APC [33,34,35,36]. More recently, we also expanded on this work using both Protac^®^ and exogenous APC. APCr was observed in SLE independently of aPL positivity, while patients with thrombosis tended to exhibit APCr to both reagents [10].

APCr assessed with the CAT analyser is not in widespread use due to limitations including high inter-laboratory variability, poor standardisation, lack of appropriate quality control materials [12,13,37], and differences in concentrations of APC, tissue factor and phospholipid vesicles. These problems make comparisons between different studies and the implementation into routine practice difficult [32,38].

While efforts have been made to improve the performance of the CAT analyser by the introduction of reference plasma for normalisation of results that reduces the inter-laboratory and inter-assay variability [11,12,37,39], proper standardisation of the method and its implementation in routine daily care remains an issue. Recent work by Douxfils et al. showed that by implementing a validated and standardised method, using commercially available reference plasma and quality control samples [17], and normalising APCr [40], steps could be made towards implementing APCr TG in routine practice as a predictive biomarker [10,11,12,13,17,30,32,33,34,35,36,37,38,39,40]. More recently, ISTH provided further guidance and recommendations for (pre)analytical steps when standardising the TG assay aiming to harmonise differences between methods and laboratories [41].

In contrast to the CAT analyser, the ST-Genesia^®^ is a new analyser for the assessment of TG, with a fully automated and standardised system aimed to introduce TG into the clinical routine. This analyser uses dedicated reagents, calibrators and internal quality controls and has been shown that it can achieve improved inter-experimental precision with the use of a reference plasma [19]. It also showed good inter-assay precision with the use of internal quality controls [42].

Our study showed that % inhibition of ETP with the ST-Genesia^®^ was significantly lower in both APS and SLE patients (mixed on and off treatment) when compared to thrombotic controls. This was not affected by anticoagulation treatment or by mixing patient plasma 50:50 with pooled normal plasma as sub-group analysis revealed that APCr for both thrombotic APS and thrombotic SLE patients on warfarin anticoagulation remained significantly higher compared to thrombotic controls. These results suggest a prothrombotic phenotype in thrombotic APS and SLE patients, in agreement with previous studies [43].

Previous assessment of the ST-Genesia^®^ and the CAT analyser showed good agreement between most but not all of the TG parameters measured [18]. One study showed limited bias between the ST-Genesia^®^ and the CAT in anticoagulated samples [42], but a different study identified significant differences in lag time, time to peak and ETP in healthy controls [44]. In addition, in patients with cirrhosis, although the ST-Genesia^®^ correctly identified patients with hypercoagulability who had been identified with the CAT analyser, others were missed [45]. Similarly, in patients undergoing a liver transplant, the CAT and the ST-Genesia^®^ provided very different results [20], suggesting that the two systems are not comparable.

In agreement with the above studies, we found poor agreement between the two analysers in APS, fair in SLE and no agreement in thrombotic control patients with a small bias in APCr values regardless of the TG method used. Agreement between the analysers remained low regardless of the method even after APS and SLE patients were sub-categorised according to clinical phenotype and aPL status. Our findings suggest that APCr evaluation with the two analysers is not comparable despite the similar methodology used.

Furthermore, differences in APCr were also identified between the three methods used in both APS and SLE patients highlighting differences in the mechanism leading to APCr. APCr with both the ST Genesia and the CAT using Protac^®^, but not the CAT using rhAPC, was significantly greater in triple antiphospholipid antibody (aPL) APS patients compared to double/single aPL patients and in thrombotic SLE patients compared to non-thrombotic SLE patients. Both methods assess the integrity of the mechanism of protein C activation compared to rhAPC, which assess the ability of the plasma to resist the anticoagulant action of exogenous provided APC. These results suggest that assessing the integrity of the endogenous protein C activation mechanism might be more sensitive in detecting differences in APCr between different clinical phenotypes and also between different aPL subtypes as both TG methods are based on activation of endogenous protein C. It might indicate that in these patients, the endogenous mechanism of activation of protein C might be defective and confirm that the use of similar TG methods might result in a higher degree of agreement between the two analysers. This could potentially be a useful tool in identifying patients at higher risk of thrombosis and in further delineating the differences between different clinical subtypes and of possible clinical significance that deems further investigation.

A novel observation of our study is that the ST-Genesia^®^ identified a significantly lower % inhibition of ETP (greater APCr) in PM compared to thrombotic APS patients that failed to reach significance with the CAT analyser for either of the two methods. The TG methods are clearly not interchangeable. In our previous studies, we have shown that there was not always complete agreement between the presence of antibodies against protein C and APCr measured by the CAT system with Protac^®^ or rhAPC, indicating differences in the TG methodology might result in different final results [9,10]. The discrepancies between methods observed in the current study could be explained by different patients having varying populations of substances that interfere with the TM catalysis of protein C activation; antibodies that block Protac^®^ cleavage of protein C; and antibodies that block APC function. It could also suggest that not only differences in the method used but also differences in the reagent composition and analyser data handling might also have affected outcomes. This could be due to a number of reasons: the concentration of TF used by the ST-Genesia^®^ ST-Genesia^®^ is unknown (the manufacturers state that it contains a medium tissue factor concentration) and might be different to that used with the CAT analyser (approximately 5 pM); the use of different reagents for the assessment of APCr (TM, rhAPC and Protac^®^) might also have played a role; other contributing factors might be the reaction conditions and the fact that although both analysers rely on the same principle, they use different calibration procedures and different software for analysis of the results. As stated above, for patients on warfarin anticoagulation, APCr assays were performed using samples mixed 50:50 with pooled normal plasma to correct for possible factor deficiencies introduced by anticoagulation. However, this is also a limitation of our study as the addition of pooled normal plasma could dilute the effect of certain antibodies, including aPL, and could introduce other variables into the system that could explain some of the differences between our study and others [46].

Further studies are required to establish the exact reasons for the differences in results between the two analysers in these patients, and disclosure of the TF and TM concentration by the manufacturer might be critical for this.

In conclusion, despite the ST-Genesia^®^ and the CAT analyser having a broadly similar methodology, an agreement was only poor to fair in patients with APS and SLE with the results not interchangeable and with no clear indication of which analyser or method gives a true reflection of hypercoagulability and greater APCr in these patients. The ST-Genesia^®^ ST-Genesia^®^ offers some clear advantages over the CAT analyser, including full automation, easier to track reagents and results for accreditation and documentation purposes that could be a benefit for a clinical laboratory or a clinical trial. However, neither method can be used as the gold standard. Measurement of TG with the addition of TM might provide a more sensitive assessment of coagulation capacity and could aid in highlighting differences in APCr between different clinical phenotypes in APS and SLE patients as TM is more physiological than Protac^®^. Additional studies are needed using both analysers to establish the effectiveness of each analyser in predicting clinical thrombotic events and, therefore, its potential use for better management of these patients. Each laboratory would be advised to establish its own reference range and performance criteria for each analyser.

## Figures and Tables

**Table 1:** APCr prevalence and categorical agreement between ST-Genesia^®^ and the CAT analyser in APS, SLE, and thrombotic control patients.

	APCr Prevalence *n* (%) (Median, 95% Confidence Intervals)			Agreemen			
Patients	ST-Genesia^®^ ™	CAT (rhAPC)	CAT (Protac^®^)	*n*(%) ST-Genesia^®^/CAT (rhAPC)	K-Coefficient	*n*(%) ST-Genesia^®^/CAT (Protac^®^)	K-Coefficient
APS (*n* = 106)	57(53.8%) (45.7%, 39.6–55.5%)	61 (57.5%) (53.6%, 48.9–58.0%)	67 (63.2%) (46.8%, 43.3–53.9%)	35 (56.8%)	0.16 Poor	44 (65.9%)	0.20 Poor
SLE (*n* = 53)	27 (50%) (49.1%, 45.2–58.5%)	32 (59.3%) (51.8%, 48.2–60.6%)	38 (70.4%) (50.9%, 44.6–56.6%)	21 (68.5%)	0.37 Fair	21 (64.8%)	0.29 Fair
Thrombotic controls (*n* = 36)	3 (8.3%) (66.3%, 61.7–72.3%)	6 (16.7%) (69.5%, 65.4–74.7%)	5 (13.9%) (69.9%, 67.5–76.6%)	0	−0.12 No	0	−0.12 No

Legend: APCr prevalence is presented as *n* (%) for the number of patients identified with APCr with each method, as well as median and 95% confident intervals. For the agreement, *n* represents the number of patients where the results were in agreement. APS: antiphospholipid syndrome; SLE: systemic Lupus erythematosus; TM: thrombomodulin; rhAPC: recombinant human activated protein C.

**Table 2:** APCr prevalence and categorical agreement between ST-Genesia^®^ and the CAT analyser in APS and SLE patients stratified according to antiphospholipid antibody status and thrombotic status.

		APCr Prevalence *n* (%) (Median, 95% Confidence Intervals)			Agreement			
Patients		ST-Genesia^®^ (TM)	CAT (rhAPC)	CAT (Protac^®^)	*n* (%) ST-Genesia^®^/CAT (rhAPC)	K-Coefficient	*n* (%) ST-Genesia^®^/CAT (Protac^®^)	K-Coefficient
APS	Thrombotic APS (*n* = 83)	42 (50.6%) (45.7%, 39.6–55.5%)	48 (57.8%) (54.3%, 42.057.5%)	49 (59.0%) (48.8%, 36.8–59.0%)	34 (59.0%)	0.22 Fair	33 (45.2%)	0.13 Poor
	Pregnancy Morbidity APS (*n* = 23)	15 (65.2%) (36.9%, 21.9–49.0%)	13 (56.5%) (46.3%, 36.177.0%)	18 (78.3%) (41.5%, 21.2–55.9%)	11 (48.2%)	0.17 Poor	14 (55.6%)	0.16 Poor
	Triple aPL positive (*n* = 15)	10 (66.6%) (37.1%, 24.7–60.5%)	12 (80.0%) (52.3%, 40.8–61.0%)	14 (93.3%) (38.4%, 23.0–52.1%)	9 (80.0%)	0.53 Moderate	11 (80.0%)	0.41 Moderate
	Double aPL positive (*n* = 36)	16 (44.4%) (52.3%, 43.2–59.4%)	21 (58.3%) (51.0%, 37–65.4%)	21 (58.3%) (45.7%, 35.6–66.2%)	12 (64.0%)	0.22 Fair	15 (70.0%)	0.37 Fair
	Single aPL positive (*n* = 32)	16 (50.0%) (52.1%, 40.7–60.6%)	18 (56.2%) (49.0%, 34.6–68.6%)	22 (68.8%) (47.5%, 32.7–70.3%)	9 (48.5%)	0.13 Poor	12 (47.2%)	0.19 Poor
SLE	aPL positive SLE (*n* = 34)	17 (50%) (45.6%, 40.4–58.5%)	18 (52.9%) (56.1%, 48.669.9%)	18 (52.9%) (58.8%, 44.7–68.7%)	11 (47.5%)	0.09 Poor	12 (54.5%)	0.08 Poor
	aPL negative SLE (*n* = 20)	9 (45.0%) (56.0%, 43.2–66.5%)	10 (50.0%) (57.8%, 34.8–90.1%)	9 (45.0%) (65.5%, 51.6–76.5%)	3 (35.0%)	0.20 Poor	3 (35.0%)	0.20 Poor
	Thrombotic SLE (*n* = 16)	10 (62.5%) (42.7%, 23.4–55.1%)	9 (56.2%) (56.1%, 45.6–69.7%)	10 (62.5%) (56.1%, 45.6–69.7%)	8 (64.8%)	0.27 Fair	8 (68.8%)	0.31 Fair
	Non thrombotic SLE (*n* = 37)	17 (45.9%) (57.8.4%, 48.0–79.1%	19 (51.4%) (57.8%, 47.9–79.1%)	17 (45.9%) (68.7%, 58.4–76.5%)	8 (47.4%)	0.06 Poor	8 (47.4%)	0.06 Poor

APCr prevalence is presented as *n* (%) for the number of patients identified with APCr with each method as well as median and 95% confident intervals. For the agreement *n* represents the number of patients where the results were in agreement. aPL: antiphospholipid antibodies.

## Data Availability

The data presented in this study are available upon reasonable request from the corresponding author.

## Supplementary Materials

The following supporting information can be downloaded at: https://www.mdpi.com/article/10.3390/jcm11010069/s1, Table S1. Demographic, clinical characteristics and treatment of thrombotic controls and APS patients, Table S2. Demographic, clinical characteristics and treatment of patients with SLE, Figure S1. Bland-Altman graphs for the agreement in APCr between ST-Genesia^®^ and the CAT analyser with rhAPC (left panel) and Protac^®^ (right panel) in A. APS patients B. SLE patients and C. thrombotic controls, Figure S2. Agreement in APCr between the ST-Genesia^®^ in the presence of TM and the CAT analyser with rhAPC (left panel) and Protac^®^ (right panel) in APS patients stratified according to clinical phenotype and aPL status, Figure S3. Bland-Altman graphs for the agreement in APCr between ST-Genesia^®^ with TM and the CAT analyser with rhAPC or Protac^®^ in APS patients stratified according to clinical phenotype and aPL status, Figure S4. Agreement in APCr between the ST-Genesia^®^ and the CAT analyser with rhAPC (left panel) and Protac^®^ (right panel) in SLE patients stratified according to aPl status and thrombotic history, Figure S5. Bland-Altman graphs for the agreement in APCr values between ST-Genesia^®^ with TM and the CAT analyser with rhAPC and Protac^®^ in patients with SLE stratified according to aPL status and thrombotic history.
