# Supplementary material for: Comparison of Acquired Activated Protein C Resistance, Using the CAT and ST-Genesia® Analysers and Three Thrombin Generation Methods, in APS and SLE Patients
Source: J Clin Med. 2021 Dec 23;11(1):69. doi: 10.3390/jcm11010069 (PMC8745056; doi:10.3390/jcm11010069)
Supplement: Supplementary file 1 [file jcm-11-00069-s001.zip › jcm-1521780-supplementary.pdf]

Supplementary information

Table S1. Demographic, clinical characteristics and treatment of thrombotic controls and APS patients.

Diagnosis	Thrombotic controls (n = 36)	All APS (n = 105)	Thrombotic APS Venous or Arterial (n = 83)	Pregnancy morbidity APS (n=22)
Age, mean years ± SD	49.1 (±15.1)	47.2± (14.7)	51.3 (± 18.4)	44.1 (± 6.8)
Sex, male/female, n	14/22	25/81	25/58	0/23
VTE only (DVT or PE), n (%)	32 (88.9)	55 (52.4)	51 (61.4)	4
AT only, n (%)	0 (0)	15 (14.2)	13 (15.7)	2
Severe thrombotic phenotype*, n (%)	4(11.1)	21(20)	19 (22.9)	2 (9.1)
VTE + AT, n (%)	2 (5.6)	11 (10.5)	11 (13.3)	2 (9.1)
Recurrent venous events, n (%)	2 (5.6)	10 (9.5)	8 (9.6)	
Pregnancy morbidity only (%)		14 (13.2)		14(63.6)
Pregnancies		63	7	56
Life births	12	22	7	15
Total APS-related PM	12	41	N/A	41
<10 weeks gestation	N/AN/A	33		33
>10 weeks gestation		8		8
APS categories ‡, n (%)				
I	N/A	64 (61.0)	51 (61.4)	13 (59.1)
/Double aPL +/Triple aPL +		45(42.8)/19 (18.1)	36 (43.4)/15 (18.1)	9 (40.9)/4 (18.2)
IIa		16 (15.2)	12 (14.5)	4 (18.2)
IIb		11 (10.5)	7(8.5)	4 (18.2)
IIc		14 (13.3)	13(15.6)	2 (9.1)
Medication				
Warfarin				
Target INR				
2.0-3.0, n (%)	32 (88.9)	55(52.4)	51 (61.4)	4 (18.2)
3.0-4.0, n (%)	4 (11.1)	36 (34.3)	32 (38.6)	4 (18.2)
Aspirin		3 (2.9)		3 (13.6)
No medication		12 (10.5)		12 (50.0)
aβ2GPI IgG (GPU) cut off 20 GPU	N/A	61.3 (14.0-132)	69.8 (19.7-132)	52.7 (14.0-87.4)
aβ2GPI IgM (MPU) cut off 20 MPU	N/A	21.2 (2.1-101)	23.1 (2.1-101)	19.2 (3.2-45.7)
aCL IgG (GPLU) cut off 20 GPLU	N/A	44.5 (3.7-144)	27.8 (3.7-142)	61.2 (12.9-144)
aCL IgM (MPLU) cut off 20 MPLU	N/A	39.9 (6.0-132)	51.3 (14.6-132.2)	28.6 (6.0-98.0)
LA, n (%)	N/A	90 (85.7)	76 (90.5)	14 (63.6)

aB2GPI: anti-beta2 glycoprotein I antibodies; aCL: cardiolipin antibodies, LA: lupus anticoagulant. *Severe thrombotic phenotype refers to patients with recurrent thromboembolic episodes despite therapeutic anticoagulation or patients with both venous thromboembolism (VTE) and arterial thrombosis (AT) .‡APS categories: I, IIa, IIb, IIc based on [22].

Table S2. Demographic, clinical characteristics and treatment of patients with SLE.

SLE PATIENTS	All SLE patients (n=53)	SLE aPL with thrombosis (n=16)	SLE aPL no thrombosis (n=20)	SLE no aPL no thrombosis (n=17)
Sex, male/female, n	7/47	4/12	5/15	3/14
Age, years (±STV)	45.6 (±12.2)	48.1 (±15.9)	46.9(±11.8)	44.5(±8.5)
**SLE manifestations (history)**				
Rash, n (%)	32 (60.4)	9 (56.3)	12 (60)	11 (64.7)
Photosensitivity, n (%)	18 (34)	5 (31.3)	7 (35)	6 (35.3)
Alopecia, n (%)	14 (26.4)	4 (25)	4 (20)	6 (35.3)
Oral ulcers, n (%)	10 (18.9)	5 (31.3)	3 (15)	2 (11.8)
Joint involvement, n (%)	42(79.2)	14 (87.5)	13 (65)	15 (88.2)
Serositis, n (%)	13 (24.5)	4 (25)	5 (25)	4 (23.5)
Renal involvement, n (%)	21 (39.6)	5 (31.3)	6 (30)	10 (58.8)
NPSLE, n (%)	11 (20.8)	5 (31.3)	3 (15)	3 (17.6)
Low C3 (normal range 0.9-1.8 g/l) and/or C4 (normal range 0.1-0.4 g/l): n (%)	29 (54.7)	7 (43.8)	10 (50)	12 (70.6)
aDNA (normal range <50 IUml): n (%)	37 (69.8)	12 (75)	12 (60)	12 (70.6)
**Patients with thrombosis (history)**				
Arterial only: n (%)	7 (13)	6(37.5)	0 (0)	0 (0)
Venous only (DVT or PE): n (%)	7 (13)	6(37.5)	0 (0)	0 (0)
Severe thrombotic phenotype**, n (%)<br>Arterial + Venous: n (%)	4 (7.4)<br>2 (3.7)	4 (25)<br>2 (12.5)	0 (0)<br>0 (0)	0 (0)<br>0 (0)
Recurent venous events: n (%)	2 (3.7)	2 (12.5)	0 (0)	0 (0)
**Disease activity at sample collection**				
Total BILAG score: median (95% centile)	2 (1-4)	2 (1-8)	3 (1-8)	1 (1-9)
Any BILAG “A”, “B” or “C”: n (%)	45 (83.3)	13 (81.3)	17 (85)	15 (83.3)
Any BILAG “A” or “B”: n (%)	19 (35.2)	5 (31.3)	8 (40)	6 (33.3)
Any BILAG “A” : n (%)	3 (5.6)	2 (6.3)	1 (5)	0(0)
SLEDAI-2K: median (95th centile)	2 (1-5)	4 (1-6)	2 (1-5)	2 (1-5)
Anti-platelet therapy, n (%)	7 (13.0)	2 (12.5)	3 (15)	2 (11.1)
Aspirin, n (%)	5 (9.3)	1 (6.3)	3 (6)	1(5.5)
Clopidrogrel, n (%)	2 (3.7)	1 (6.3)	0 (0)	1 (5.5)
Anticoagulant therapy, n (%)	15 (27.8)	15 (93.8)	0 (0)	0 (0)
VKA, n (%)<br><br>Target INR<br>2.0-3.0, n (%)<br>3.0-4.0, n (%)	15 (27.8)<br><br>5 (9.3)<br>10 (18.5)	15 (93.8)<br><br>5 (31.3)<br>10 (62.5)	0 (0)<br><br>0 (0)<br>0 (0)	0 (0)<br><br>0 (0)<br>0 (0)
aPL status				
aβ2GPI IgG (GPU)	22.1 (1.0-72.0)	35.4 (7.0-72.0)	28.5 (8.0-52.4)	2.5 (1.0-4.3)
aβ2GPI IgM (MPU)	15.1 (2.5-69.2)	21.2 (4.1-65.7)	19.2(5.0-69.2)	4.2 (2.5-6.1)
aCL IgG (GPLU)	14.9 (0.8-65.4)	23.8 (5.2-65.4)	18.9 (6.2-26.2)	2.1 (0.8-5.3)
aCL IgM (MPLU)	13.1 (0.4-81.0)	17.6 (5.9-41.0)	20.5 (5.1-81.0)	1.3 (0.4-3.2)
LA, n (%)	30 (55.6)	13 (81.3)	15 (75)	0 (0)
APS Category I: n (%)	29 (53.7)	13 (81.3)	16 (80)	0 (0)
Double aPL positive: n (%)	16 (29.6)	7 (43.8)	9 (45)	0 (0)
Triple aPL positive: n (%)	13 (24.1)	6 (37.5)	7(35)	0 (0)
Category IIa: n (%)	4 (7.4)	2 (12.5)	2 (10)	0 (0)
Category IIb: n (%)	2 (3.7)	1 (6.3)	1(5)	0 (0)
Category IIc: n (%)	1 (1.85)	0 (0)	1(5)	0 (0)

aPL: antiphospholipid antibodies, aB2GPI: anti-beta2 glycoprotein I antibodies; anti-DNA: anti-DNA antibodies, aCL: cardiolipin antibodies, LA: lupus anticoagulant, SLE: systemic lupus erythematosus.

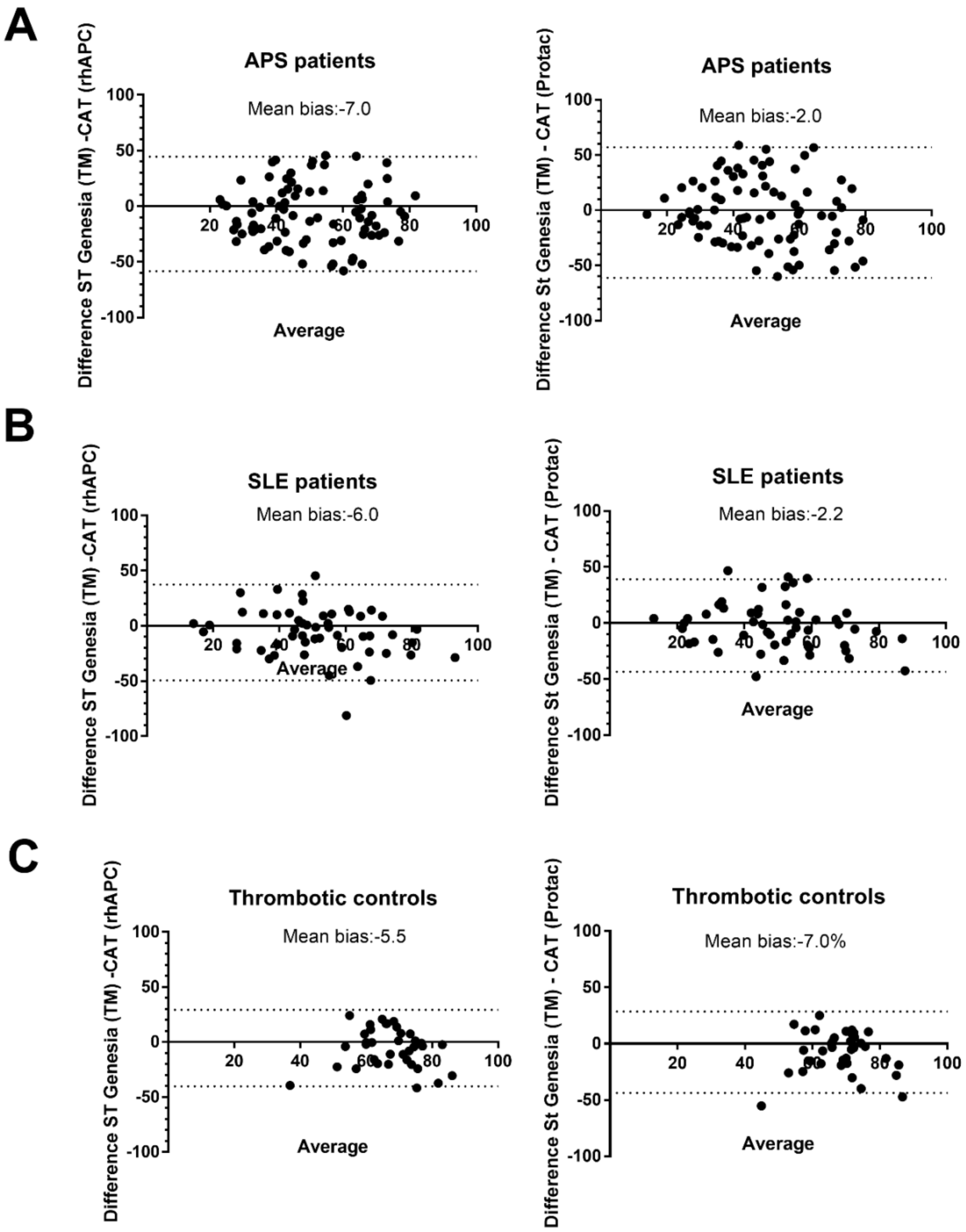

Figure S1. Bland-Altman graphs for the agreement in APCr between ST-Genesia® with and the CAT analyser with rhAPC (left panel) and Protac® (right panel) in **A.** APS patients **B.** SLE patients and **C.** thrombotic controls. Horizontal broken lines indicate the 95% limits of agreement.

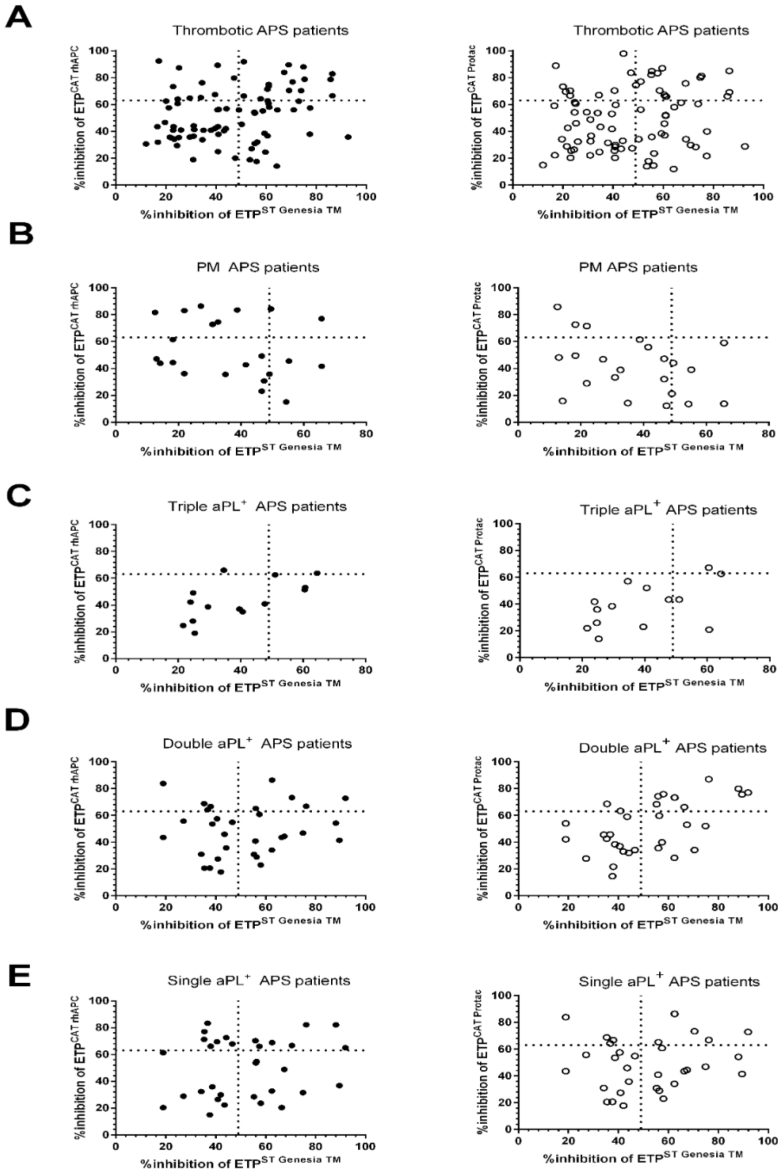

Figure S2. Agreement in APCr between the ST-Genesia® in the presence of TM and the CAT analyser with rhAPC (left panel) and Protac® (right panel) in APS patients stratified according to clinical phenotype and aPL status. A. thrombotic, B.pregnancy morbidity (PM), C. triple aPL positive (+), D. double aPL + and E. single aPL+ APS patients,

Horizontal broken line represents the cut off value for APCr with the ST-Genesia® (49%) and vertical dotted line in the left panel represents the APCr cut off value of 56% for the CAT with rhAPC and in the right panel the APCr cut off value of 63% for the CAT with Protac®. All cut off values were defined as below the 99th centile of NC. PM: pregnancy morbidity, aPL: antiphospholipid antibody

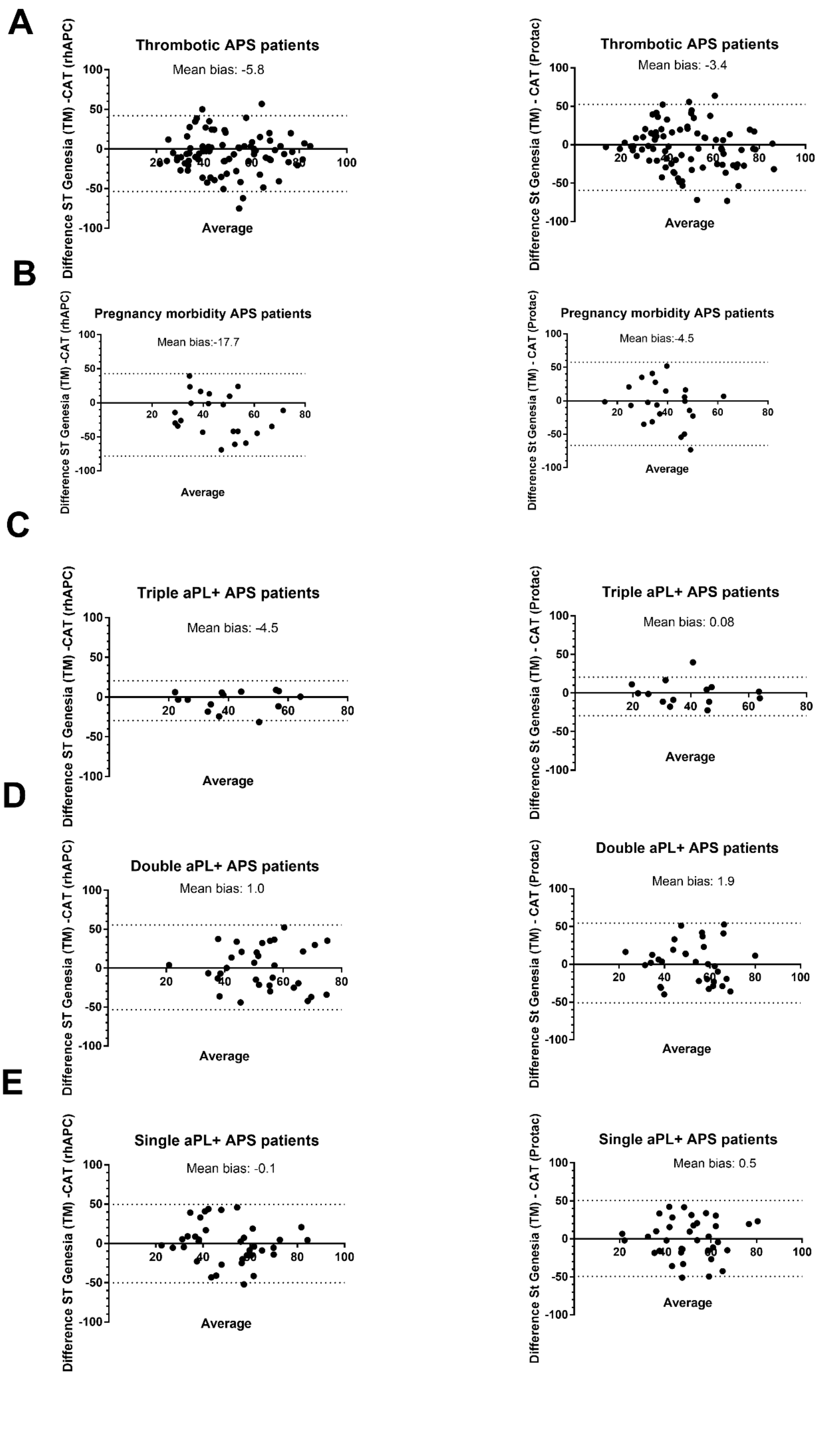
A
Thrombotic APS patients
Mean bias: -5.8
Difference ST Genesia (TM) -CAT (rhAPC)
Average
Thrombotic APS patients
Mean bias: -3.4
Difference St Genesia (TM) - CAT (Protac)
Average
B
Pregnancy morbidity APS patients
Mean bias:-17.7
Difference ST Genesia (TM) -CAT (rhAPC)
Average
Pregnancy morbidity APS patients
Mean bias:-4.5
Difference St Genesia (TM) - CAT (Protac)
Average
C
Triple aPL+ APS patients
Mean bias: -4.5
Difference ST Genesia (TM) -CAT (rhAPC)
Average
Triple aPL+ APS patients
Mean bias: 0.08
Difference St Genesia (TM) - CAT (Protac)
Average
D
Double aPL+ APS patients
Mean bias: 1.0
Difference ST Genesia (TM) -CAT (rhAPC)
Average
Double aPL+ APS patients
Mean bias: 1.9
Difference St Genesia (TM) - CAT (Protac)
Average
E
Single aPL+ APS patients
Mean bias: -0.1
Difference ST Genesia (TM) -CAT (rhAPC)
Average
Single aPL+ APS patients
Mean bias: 0.5
Difference St Genesia (TM) - CAT (Protac)
Average

Figure S3. Bland-Altman graphs for the agreement in APCr values between ST-Genesia® with TM and the CAT analyser with rhAPC or Protac® in APS patients stratified according to clinical phenotype and aPL status: A. thrombotic and B. pregnancy morbidity C. triple D. double E. single aPL positive. Horizontal broken lines indicate the 95% limits of agreement.

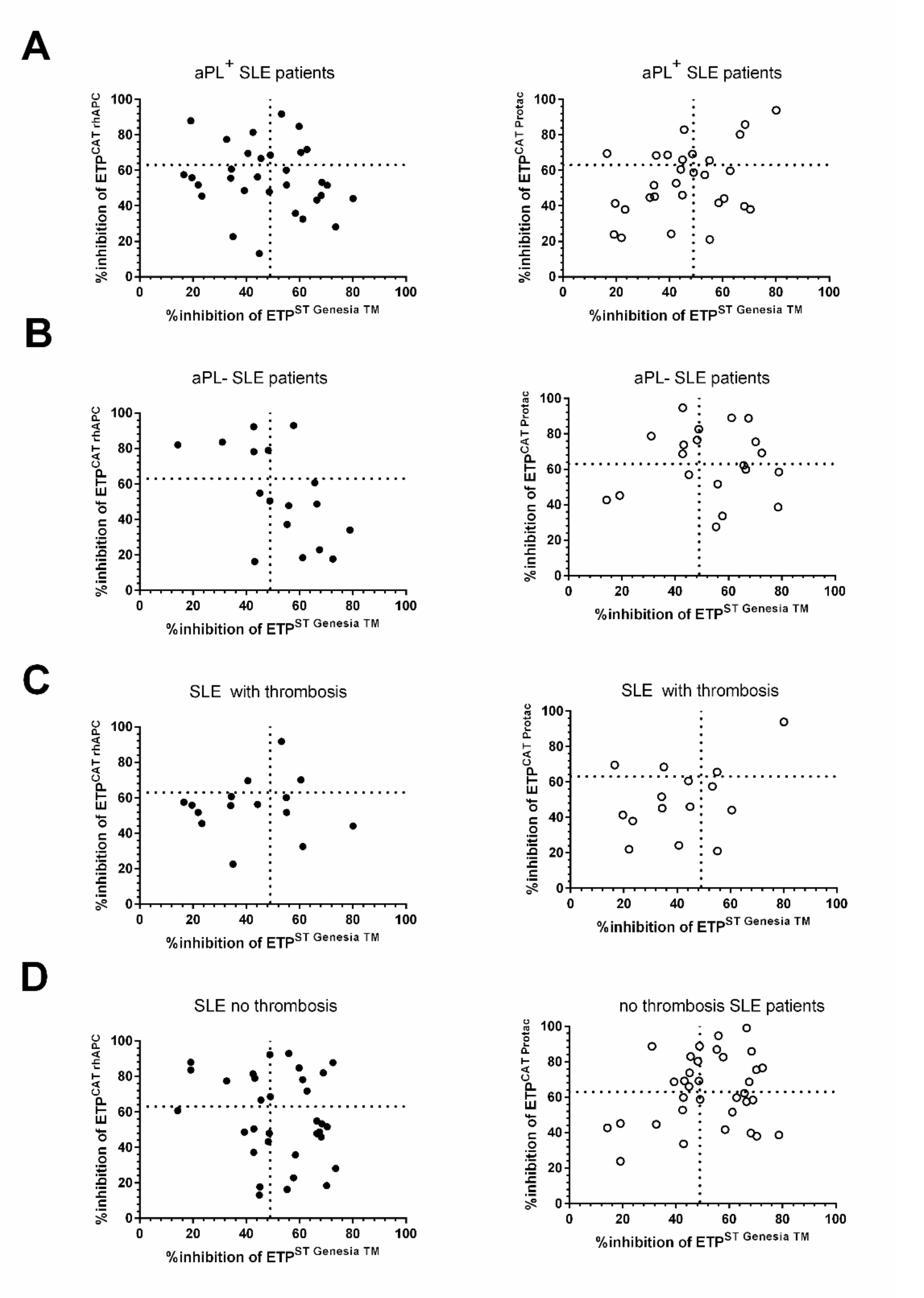

Figure S4.

Agreement in APCr between the ST-Genesia® and the CAT analyser with rhAPC (left panel) and Protac® (right panel) in SLE patients stratified according to aPl status and thrombotic history: A. aPL positive, B. aPL negative (-) and according to into C. thrombotic and D. non thrombotic.

Horizontal broken line represents the cut off value for APCr with the ST-Genesia® (49%) and vertical dotted line in the left panel represents the APCr cut off value of 56% for the CAT with rhAPC and in the right panel the APCR cut off value of 63% for the CAT with Protac®. All cut off values were defined as below the 99th centile of NC.

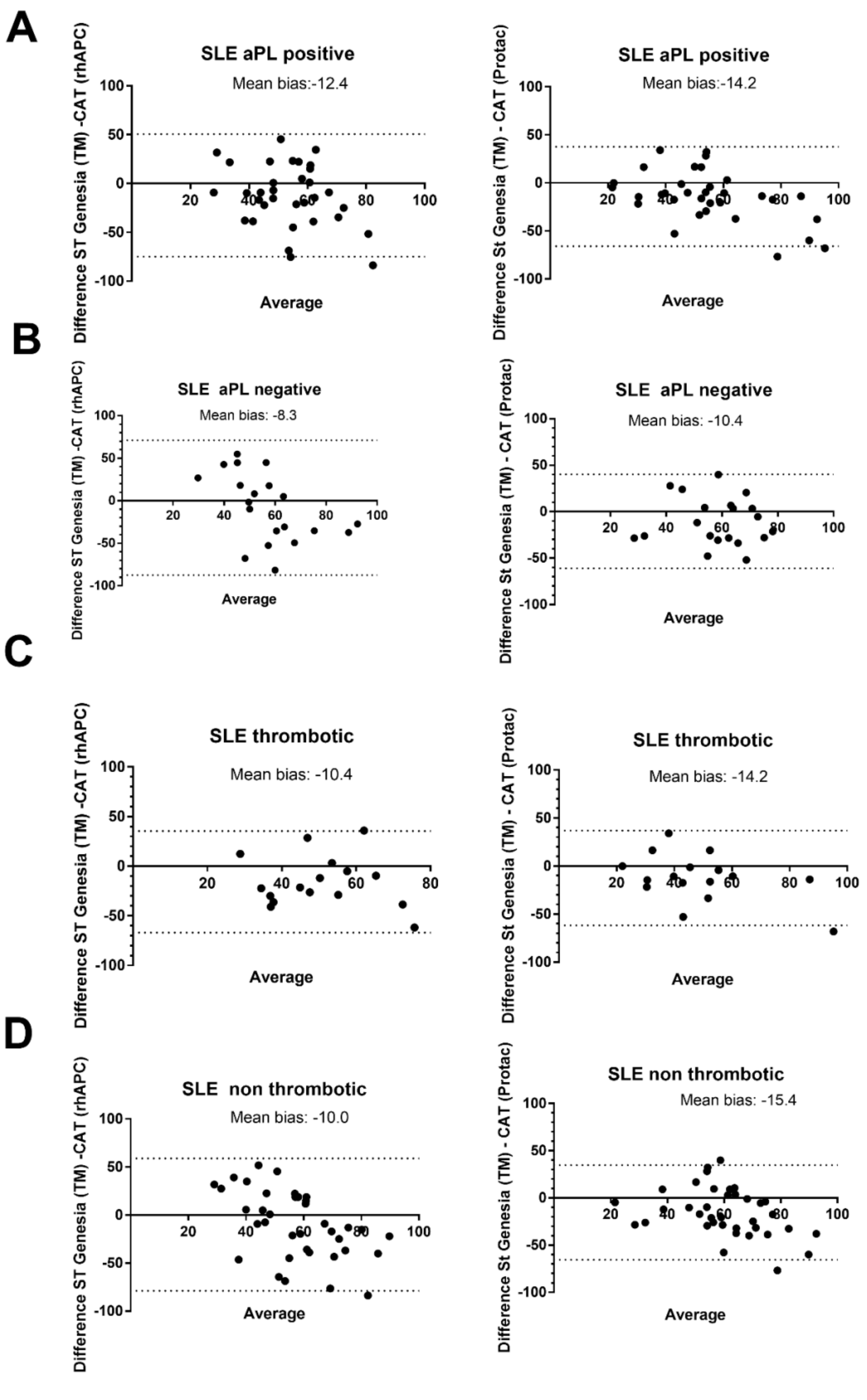

Figure S5. Bland-Altman graphs for the agreement in APCr values between ST-Genesia® with TM and the CAT analyser with rhAPC and Protac® in patients with SLE stratified according to aPL status and thrombotic history A. aPL positive B. aPL negative C. thrombotic and D. non thrombotic SLE. Horizontal broken lines indicate the 95% limits of agreement.
